# Bivalent mRNA vaccine effectiveness against COVID-19 among older adults in Japan: a test-negative study from the VENUS study

**DOI:** 10.1186/s12879-024-09035-3

**Published:** 2024-01-29

**Authors:** Yudai Tamada, Kenji Takeuchi, Taro Kusama, Megumi Maeda, Fumiko Murata, Ken Osaka, Haruhisa Fukuda

**Affiliations:** 1 Department of International and Community Oral Health, Tohoku University Graduate School of Dentistry, 4-1, Seiryo-Machi, Aoba-Ku, Sendai, Miyagi 980-8575 Japan; 2 Department of Preventive Medicine, Nagoya University Graduate School of Medicine, Aichi, Japan; 3 Division of Statistics and Data Science, Liaison Center for Innovative Dentistry, Tohoku University Graduate School of Dentistry, Miyagi, Japan; 4 Department of Health Care Administration and Management, Kyushu University Graduate School of Medical Sciences, Fukuoka, Japan

**Keywords:** COVID-19, SARS-CoV-2, Omicron, Booster, Vaccination

## Abstract

**Background:**

Bivalent COVID-19 vaccines have been implemented worldwide since the booster vaccination campaigns of autumn of 2022, but little is known about their effectiveness. Thus, this study holistically evaluated the effectiveness of bivalent vaccines against infection in older adults in Japan.

**Methods:**

We adopted the test-negative design using COVID-19 test data of individuals, aged ≥ 65 years, residing in three municipalities in Japan, who underwent tests in medical institutions between October 1 and December 30, 2022. Logistic regression analyses were conducted to estimate the odds of testing positive according to vaccination status. Vaccine effectiveness was defined as (1 − odds ratio) × 100%.

**Results:**

A total of 3,908 positive and 16,090 negative results were included in the analyses. Receiving a bivalent dose in addition to ≥ 2 monovalent doses was 33.6% (95% confidence interval [CI]: 20.8, 44.3%) more effective than receiving no vaccination, and 18.2% (95% CI: 9.4, 26.0%) more effective than receiving ≥ 2 monovalent doses but not receiving a bivalent vaccination. In addition, the effectiveness peaked at 14–20 days after administration and then gradually declined over time. Furthermore, a bivalent booster dose provided 18.6% (95% CI: 9.9, 26.5%) additional protection among those vaccinated with ≥ 2 monovalent doses, in the absence of a previous infection history. However, we did not find sufficient evidence of effectiveness of bivalent vaccines among previously infected older adults.

**Conclusions:**

Bivalent vaccines are effective against COVID-19 infections among older adults without a history of infection.

**Supplementary Information:**

The online version contains supplementary material available at 10.1186/s12879-024-09035-3.

## Background

The original monovalent COVID-19 vaccines successfully provided protection against COVID-19 infection and severe disease, including hospitalization and death. However, their effectiveness has declined due to the waning of natural or vaccine conferred immunity [1–3] and the emergence of more immune-evasive virus variants. Hence, to improve protection, bivalent mRNA vaccines containing the components of the ancestral strain and the Omicron BA.1 or BA.4/5 sublineages were implemented in the autumn 2022 booster vaccination campaigns worldwide. Early reports showed moderate additional protection conferred by a bivalent booster dose [4, 5], but limited studies have assessed the vaccine effectiveness (VE) of bivalent vaccines among previously infected people [6, 7].

In Japan, bivalent vaccines against the BA.1 variant have been administered as a booster dose since September 20, 2022, to people who had received their latest vaccination at least 5 months earlier. Then, bivalent vaccines targeting BA.4/5 began to be offered from October 13, 2022, and the interval from the last vaccination was shortened to 3 months from October 21, 2022. As of the end of 2022, around 35% of the whole Japanese population and > 60% of the population aged ≥ 65 years had been vaccinated with bivalent vaccines [8]. Despite the importance of scientific literature that reflects a range of experiences from different populations, no reports have been available on the effectiveness of bivalent vaccines in Eastern countries, including Japan. Therefore, this study aimed to investigate whether bivalent vaccines are effective against COVID-19 infection among older adults and whether a bivalent booster dose provides additional protection against infection as compared to monovalent doses alone, regardless of a history of infection.

## Methods

### Study population and setting

This study was based on data from the Vaccine Effectiveness, Networking, and Universal Safety (VENUS) study [9], a multi-region community-based database project aimed at evaluating the safety and effectiveness of vaccines in real-world settings. As of May 2023, the VENUS study had collected data from 13 municipalities in Japan. Of these municipalities, three provided the following health-related data covering the study period: healthcare claims data of enrollees of the two insurance systems used in Japan (National Health Insurance and Latter-Stage Older Persons Health Care System), Health Center Real-time Information-sharing System on COVID-19 (HER-SYS) data [10], and Vaccination Record System (VRS) data [11]. These municipalities are located across Japan (Kanto, Chubu, and Chugoku regions) and have resident populations ranging from 220,000 to 410,000 (older adults aged ≥ 65 years in the three municipalities comprised approximately 0.8% of the total population aged ≥ 65 years in Japan). In the VENUS study, a unique research ID was assigned to each resident by the data managers, and each data point was linked at the individual level. In addition to the VENUS study’s profile paper [9], given that the VENUS study is a subproject of the Longevity Improvement & Fair Evidence (LIFE) study, the details of the data collection procedures and the database construction are available in the LIFE study’s profile paper [12].

In this test-negative study [13], using the data of participants who underwent COVID-19 tests in medical institutions as administrative inspections between October 1 and December 30, 2022, we compared the vaccination status of tested-positive participants with that of tested-negative participants to estimate the effectiveness of COVID-19 vaccines. The test date and type—polymerase chain reaction (PCR) test or antigen test—were identified using the corresponding procedure codes in the healthcare claims data. The beginning date of the study period was determined because at least 7 days—sufficient time to acquire immunity by the booster vaccination [14]—had passed from the date of initiating bivalent vaccine administration in the participating municipalities. During the study period, COVID-19 tests were mainly available at mass testing centers, pharmacies, home (using self-testing kits), and medical institutions. At mass testing centers and pharmacies, people without COVID-19-like symptoms (e.g., fever) could receive COVID-19 tests for free or at a lower cost whenever they want. Approved self-testing kits were available at pharmacies and granted online shops. For people with COVID-19-like symptoms, especially if they were at a high-risk population such as aged ≥ 65 years, they were highly recommended to receive COVID-19 tests at medical institutions. At medical institutions, COVID-19 tests were conducted with public support for their testing fees under the Act on the Prevention of Infectious Diseases and Medical Care for Patients with Infectious Diseases (Infectious Diseases Control Act). PCR and antigen tests were both used to make a definitive diagnosis and there were not great differences in their eligibility and accessibility.

**Fig. 1:**
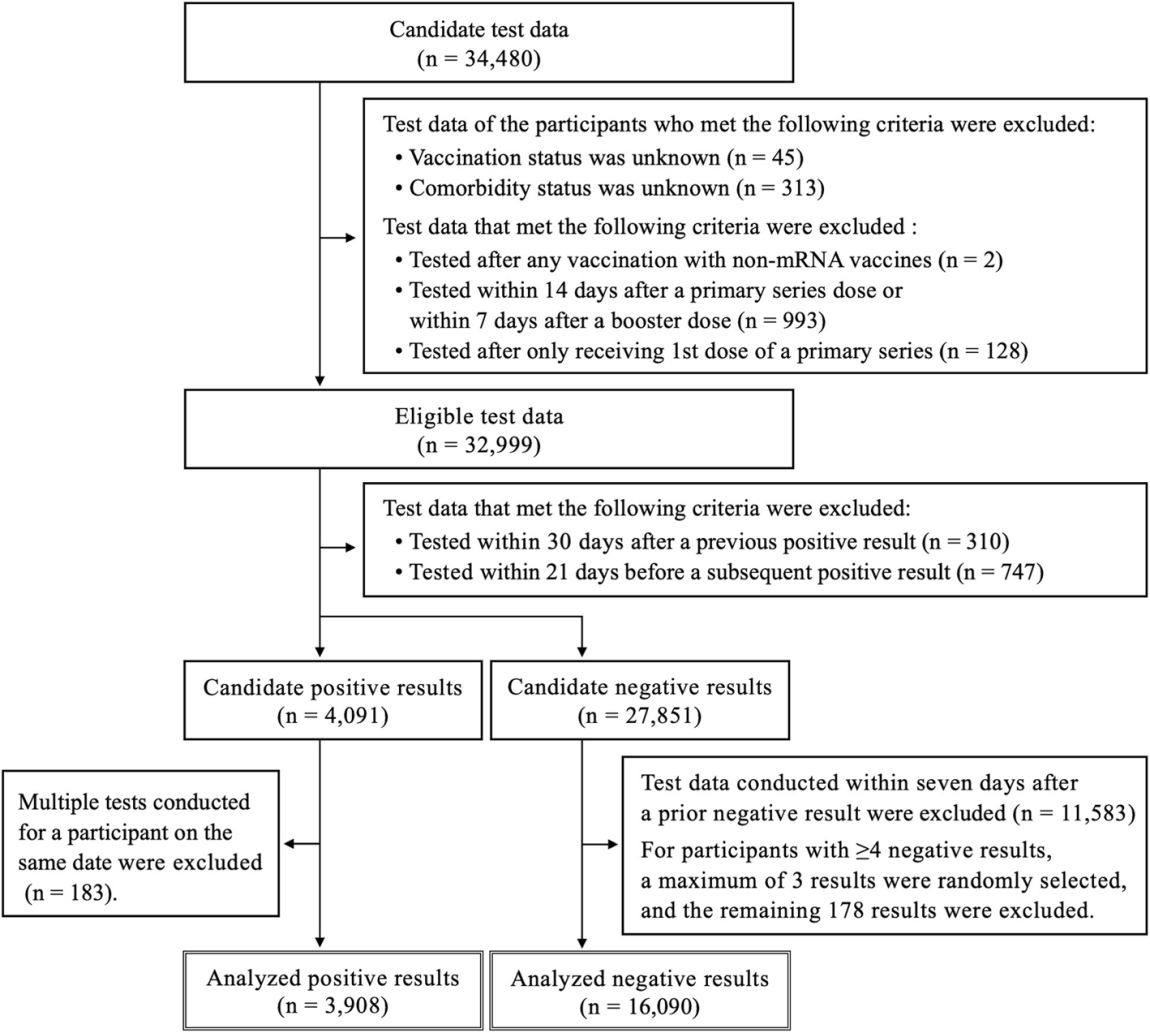
Diagram of the analyzed test results selection

For the analysis, we extracted 34,480 candidate test data, with public-expense support for testing fees, collected during the study period. After excluding test data of participants whose vaccination (*n* = 45) or comorbidity (*n* = 313) status were unknown, we adopted the following test results selection criteria, according to previous studies [15–18]: (1) Test results obtained after any vaccination with non-mRNA (e.g., adenoviral vector or protein subunit) vaccines were excluded, to ensure estimation of the effectiveness of the mRNA vaccines (*n* = 2). (2) Test results obtained within 14 days after a primary series dose or 7 days after a booster dose were excluded (*n* = 993), because these intervals are needed to acquire immunity and given the possibility of reactogenicity. (3) Test results of participants who only received their first dose of the primary series were excluded (*n* = 128), as a limited number of participants were in the middle of the primary series. (4) Results of participants with tests conducted within 30 days after a previous positive result were excluded (*n* = 310), due to the possibility of a single prolonged illness episode. (5) For participants with multiple positive results obtained with tests conducted on the same date, only one result was included (183 results were excluded). (6) Negative results to a test conducted within 21 days before a subsequent positive result were excluded (*n* = 747), due to the possibility of false negatives. (7) Any negative results obtained within 7 days after a prior negative result were excluded (*n* = 11,583), as they could reflect a single prolonged illness episode. (8) For participants with more than four negative results, a maximum of three negative results were randomly selected (178 results were excluded). The details of the selected test results are presented in a flow diagram (Fig. 1).

### Outcome assessment

The primary outcome of this study was COVID-19 infection as identified by the existence of records in the HER-SYS data. During the study period, people aged ≥ 65 years who tested positive for COVID-19 were obliged to report their infection status to the public health center of the jurisdiction under the Infectious Diseases Control Act. During the reporting process, the HER-SYS was used to enter the required information (e.g., name, birth date, sex, and testing date) [10]. By integrating the HER-SYS and healthcare claims data, we identified whether the participants had received positive or negative results at the testing date. Supplemental Fig. 1 shows the trends in the number of newly confirmed COVID-19 cases among older adults aged ≥ 65 years in the three municipalities from January 1 to December 31, 2022. Around our study period, BF.5 and BA.5.2 variants were the dominant circulating variants, which consisted of more than half of the cases.

### Exposure assessment

The exposure in this study was the COVID-19 vaccination status, defined in several ways, considering whether bivalent vaccines were received, and the number of monovalent doses that were received. In Japan, when people have received COVID-19 vaccines, their demographic and vaccination-related information (e.g., name, birth date, sex, vaccine brand names, and dates of each dose) is recorded in the VRS [11]. By integrating the VRS and healthcare claims data, we assessed the participants’ vaccination status at the testing date. In this assessment, we regarded participants as unvaccinated if they were not recorded in the VRS.

In Japan, the following four bivalent mRNA vaccines were available around the study period: Pfizer BNT162b2 BA.1 vaccine, Pfizer BNT162b2 BA.4/5 vaccine, Moderna mRNA-1273 BA.1 vaccine, and Moderna mRNA-1273 BA.4/5 vaccine. People could receive the bivalent dose after three months passed from receiving the latest monovalent dose (five months were required before October 20, 2022) in mass vaccination sites or medical institutions without charge. Since older adults were regarded as a high-risk population for severe illness, the bivalent vaccines were prioritized to be distributed to older adults. As a result, older adults could receive BNT162b2 BA.1 vaccine from September 20, 2022, BNT162b2 BA.4/5 vaccine from October 13, 2022, mRNA-1273 BA.1 vaccine from September 20, 2022, and mRNA-1273 BA.4/5 vaccine from November 28, 2022. Among our study participants who received the bivalent dose, 646 (17.5%) received BNT162b2 BA.1 vaccine, 2,971 (80.3%) received BNT162b2 BA.4/5 vaccine, 81 (2.2%) received mRNA-1273 BA.1 vaccine, and 4 (0.1%) received mRNA-1273 BA.4/5 vaccine (Supplemental Table 1).

### Other covariates

To assess the characteristics of the sample for analysis, the following variables were considered: sex (male or female), age group (65–69, 70–74, 75–79, 80–84, or ≥ 85 years), number of comorbidities (0–1, 2–4, or ≥ 5), COVID-19 infection history (with or without), residential municipality (City A, City B, or City C), interval since last vaccine dose, test type (PCR or antigen test), and test week (October 1–7, October 8–14, October 15–21, October 22–28, October 29–November 4, November 5–11, November 12–18, November 19–25, November 26–December 2, December 3–9, December 10–16, December 17–23, or December 24–30). Age groups were classified according to age as of September 2022. The number of comorbidities was defined by counting the number of medical conditions in the Charlson Comorbidity Index [19]. The medical conditions (17 conditions in total) were identified using the corresponding International Classification of Diseases, 10th Revision, codes in the healthcare claims data of November 2021–September 2022. Participants with records in the HER-SYS database before the testing date were regarded as having a history of infection. The HER-SYS was implemented in May 2020 and it was mandatory to register their infection status with the HER-SYS when people tested positive for COVID-19 in Japan; hence, the almost all infection cases, especially infected from May 2020 until the testing date, were considered to be precisely identified. The latest infection was considered in those with a history of multiple infections.

### Statistical analysis

For descriptive characteristics, summary statistics were constructed using the frequencies for each variable. For the primary analysis, we evaluated the VE in several settings. First, we compared the test results of the participants who received a bivalent dose in addition to ≥ 2 monovalent vaccine doses with those of unvaccinated participants (absolute VE) and those of the participants who received ≥ 2 monovalent doses but not a bivalent vaccine dose (relative VE). Second, we evaluated the absolute and relative VE according to the interval since receiving a bivalent dose (≤ 13, 14–20, 21–34, or ≥ 35 days) to assess the short-term duration of bivalent vaccine-conferred protection. Third, we evaluated the absolute and relative VE of the bivalent vaccines as stratified by a COVID-19 infection history to assess whether a bivalent booster dose provided additional protection regardless of an infection history. To assess VE, a logistic regression model with cluster-robust standard errors at the individual level was used to estimate the odds ratios (ORs) and their corresponding 95% confidence intervals (CIs) for testing positive according to vaccination status. VE was defined as (1 − OR) × 100%. In the analyses, sex, age group, number of comorbidities, infection history, residential municipality, and test week were adjusted, except for the stratified analysis according to the presence or absence of previous infection.

In addition to the primary analysis, we conducted five auxiliary analyses. First, we compared the effectiveness of BA.4/5 bivalent vaccines to that of BA.1 bivalent vaccines. In the analysis, we restricted our analytical sample to test results of the participants vaccinated with either the BA.1 or BA.4/5 bivalent vaccines between October 22 and December 30, 2022 (during the period, both the vaccines were available in Japan). Considering that the BA.1 vaccines were initiated earlier than the BA.4/5 vaccines, if the participants had received the BA.1 vaccines when the BA.4/5 vaccines were not available, their BA.1 vaccine-conferred immunity might have already decreased by the time the BA.4/5 vaccines became available. Hence, we restricted our analytical sample to avoid underestimation of the effectiveness of the BA.1 vaccines by including the participants vaccinated with the BA.1 vaccines before the BA.4/5 vaccines became available. Second, we conducted the subgroup analysis by test type (PCR or antigen tests) to assess the robustness of our results by misclassification bias due to the differences in diagnostic accuracy between PCR and antigen tests. Third, we assessed the VE using the vaccination status variable that classified “ ≥ 2 monovalent doses plus a bivalent dose” in more detail by the number of received monovalent doses before receiving the bivalent dose. Fourth, we assessed the VE using the vaccination status variable that classified “ ≥ 2 monovalent doses plus a bivalent dose” in more detail by the bivalent vaccines’ brand name. Fifth, we assessed the differences in the likelihood of hospitalization after testing positive among the tested-positive participants according to vaccination status. According to the definition used in previous studies [17, 20], we regarded hospitalizations within 7 or 14 days of testing positive as COVID-19 related-hospitalizations.

All analyses were conducted using Stata (version 17.0; Stata Corp., College Station, TX, USA). This study followed the Strengthening the Reporting of Observational Studies in Epidemiology (STROBE) guidelines.

## Results

Table 1 shows the characteristics of the 19,998 analyzed test results of 17,080 participants (age: 80.9 ± 7.7 years [mean ± standard deviation, SD], 42.3% male) according to the test results or the vaccination status. Of the test results, 80.5% were negative and 19.5% were positive. On the testing date, 5.5% of cases were unvaccinated, 4.0% had received two monovalent doses, 13.0% had received three monovalent doses, 58.9% had received four monovalent doses, and 18.5% had received two monovalent doses plus a bivalent dose. In addition, compared to the positive results, negative results tended to be found in participants who were male, were older, had more comorbidities, had a COVID-19 infection history, and lived in city B. Moreover, at the testing date, for those who had received two, three, or four monovalent doses or ≥ 2 monovalent doses plus a bivalent dose, 454.0 (SD: 96.7), 235.3 (SD: 55.7), 97.0 (SD: 28.9), or 25.5 (SD: 15.6) mean days had passed since their last dose, respectively. Of the test results, 50.6% were obtained from PCR tests. Furthermore, the prevalence of positive results per week showed an increasing trend, with 29.0% of the tests conducted on December 24–30, 2022 producing positive results.

**Table 1:** Characteristics of the analyzed test results according to test results and vaccination status

	**Analyzed test results**		** Vaccination status**				
	**Negative** (*n* = 16,090)	**Positive** (*n* = 3,908)	**Unvaccinated** (*n* = 1,107)	**2 monovalent doses** (*n* = 797)	**3 monovalent doses** (*n* = 2,608)	**4 monovalent doses** (*n* = 11,784)	** ≥ 2 monovalent doses plus a bivalent dose** (*n* = 3,702)
	n (row %)	n (row %)	n (row %)	n (row %)	n (row %)	n (row %)	n (row %)
**Test results**							
Negative	16,090 (100.0)	—	854 (5.3)	635 (3.9)	2,183 (13.6)	9,565 (59.4)	2,853 (17.7)
Positive	—	3,908 (100.0)	253 (6.5)	162 (4.1)	425 (10.9)	2,219 (56.8)	849 (21.7)
**Sex**							
Male	6,965 (81.6)	1,567 (18.4)	438 (5.1)	330 (3.9)	1,126 (13.2)	4,953 (58.1)	1,685 (19.7)
Female	9,125 (79.6)	2,341 (20.4)	669 (5.8)	467 (4.1)	1,482 (12.9)	6,831 (59.6)	2,017 (17.6)
**Age group, years**							
65–69	902 (68.2)	421 (31.8)	85 (6.4)	74 (5.6)	212 (16.0)	750 (56.7)	202 (15.3)
70–74	2,226 (75.6)	719 (24.4)	175 (5.9)	115 (3.9)	387 (13.1)	1,759 (59.7)	509 (17.3)
75–79	3,340 (78.4)	921 (21.6)	194 (4.6)	163 (3.8)	514 (12.1)	2,493 (58.5)	897 (21.1)
80–84	3,764 (81.2)	869 (18.8)	248 (5.4)	169 (3.6)	598 (12.9)	2,681 (57.9)	937 (20.2)
≥ 85	5,858 (85.7)	978 (14.3)	405 (5.9)	276 (4.0)	897 (13.1)	4,101 (60.0)	1,157 (16.9)
**Number of comorbidities**							
0–1	4,036 (73.1)	1,486 (26.9)	319 (5.8)	208 (3.8)	584 (10.6)	3,341 (60.5)	1,070 (19.4)
2–4	8,283 (81.7)	1,860 (18.3)	563 (5.6)	364 (3.6)	1,244 (12.3)	6,081 (60.0)	1,891 (18.6)
≥ 5	3,771 (87.0)	562 (13.0)	225 (5.2)	225 (5.2)	780 (18.0)	2,362 (54.5)	741 (17.1)
**Infection history**							
With	1,424 (96.9)	46 (3.1)	977 (5.3)	713 (3.8)	2,201 (11.9)	11,202 (60.5)	3,435 (18.5)
Without	14,666 (79.2)	3,862 (20.8)	130 (8.8)	84 (5.7)	407 (27.7)	582 (39.6)	267 (18.2)
**Residential municipality**							
City A	3,792 (79.4)	983 (20.6)	331 (6.9)	222 (4.6)	703 (14.7)	2,662 (55.7)	857 (17.9)
City B	4,684 (87.4)	678 (12.6)	280 (5.2)	211 (3.9)	655 (12.2)	3,230 (60.2)	986 (18.4)
City C	7,614 (77.2)	2,247 (22.8)	496 (5.0)	364 (3.7)	1,250 (12.7)	5,892 (59.8)	1,859 (18.9)
**Interval since last vaccine dose**, mean days (SD)	—	—	—	454.0 (96.7)	235.3 (55.7)	97.0 (28.9)	25.5 (15.6)
**Test type**							
PCR	8,721 (86.2)	1,391 (13.8)	575 (5.7)	399 (3.9)	1,418 (14.0)	6,136 (60.7)	1,584 (15.7)
Antigen	7,369 (74.5)	2,517 (25.5)	532 (5.4)	398 (4.0)	1,190 (12.0)	5,648 (57.1)	2,118 (21.4)
**Test week**							
1 Oct–7 Oct	1,240 (92.5)	101 (7.5)	79 (5.9)	62 (4.6)	248 (18.5)	951 (70.9)	1 (0.1)
8 Oct–14 Oct	1,019 (92.0)	89 (8.0)	51 (4.6)	48 (4.3)	178 (16.1)	819 (73.9)	12 (1.1)
15 Oct–21 Oct	1,194 (90.1)	131 (9.9)	61 (4.6)	56 (4.2)	214 (16.2)	972 (73.4)	22 (1.7)
22 Oct–28 Oct	1,134 (90.3)	122 (9.7)	69 (5.5)	46 (3.7)	187 (14.9)	918 (73.1)	36 (2.9)
29 Oct–4 Nov	1,072 (83.6)	211 (16.4)	75 (5.8)	44 (3.4)	175 (13.6)	957 (74.6)	32 (2.5)
5 Nov–11 Nov	1,149 (81.0)	269 (19.0)	58 (4.1)	45 (3.2)	202 (14.2)	1,050 (74.0)	63 (4.4)
12 Nov–18 Nov	1,344 (81.2)	311 (18.8)	94 (5.7)	71 (4.3)	223 (13.5)	1,135 (68.6)	132 (8.0)
19 Nov–25 Nov	1,265 (76.0)	400 (24.0)	92 (5.5)	61 (3.7)	224 (13.5)	1,081 (64.9)	207 (12.4)
26 Nov–2 Dec	1,341 (78.2)	374 (21.8)	103 (6.0)	75 (4.4)	205 (12.0)	987 (57.6)	345 (20.1)
3 Dec–9 Dec	1,334 (75.6)	431 (24.4)	110 (6.2)	56 (3.2)	193 (10.9)	897 (50.8)	509 (28.8)
10 Dec–16 Dec	1,288 (72.6)	485 (27.4)	94 (5.3)	76 (4.3)	186 (10.5)	821 (46.3)	596 (33.6)
17 Dec–23 Dec	1,470 (75.5)	478 (24.5)	124 (6.4)	85 (4.4)	204 (10.5)	662 (34.0)	873 (44.8)
24 Dec–30 Dec	1,240 (71.0)	506 (29.0)	97 (5.6)	72 (4.1)	169 (9.7)	534 (30.6)	874 (50.1)

Analyzed 19,998 test results were from 17,080 participants (age: 80.9 ± 7.7 years [mean ± standard deviation], 42.3% male). Since some participants received one test and some received ≥ 2 tests during the study period., our participants received a mean of 1.2 tests per participant

*Abbreviations*: *SD* standard deviation, *PCR* polymerase chain reaction

Table 2 presents the absolute and relative VE of the bivalent vaccines against infection. Compared with the unvaccinated group, the absolute VE in those who had received ≥ 2 monovalent doses plus a bivalent dose was 33.6% (95% CI: 20.8, 44.3%), while it was 18.8% (95% CI: 4.9, 30.7%) in those who had received ≥ 2 monovalent doses but not a bivalent dose. In addition, among those who had received ≥ 2 monovalent doses but not received a bivalent dose, while receiving three or four monovalent doses was approximately 20% effective, receiving two monovalent doses (i.e., completed primary series only) was 12.8% (95% CI: -11.4, 31.7%). Regarding the relative VE, receiving a bivalent dose in addition to ≥ 2 monovalent doses was 18.2% (95% CI: 9.4, 26.0%) more effective than receiving ≥ 2 monovalent doses but not receiving a bivalent dose. In addition, as compared with receiving two monovalent doses but not receiving a bivalent dose, receiving ≥ 2 monovalent doses plus a bivalent dose was 23.8% (95% CI: 6.0, 38.2%) effective.

**Table 2:** Absolute or relative VE of bivalent vaccines against infection

	**Absolute VE**^**ab**^, % (95% CI)	**Absolute VE**^**ac**^, % (95% CI)	**Relative VE**^**a**^, % (95% CI)	**Relative VE**^**a**^, % (95% CI)
**Vaccination status**				
Unvaccinated	Ref	Ref	**—**	**—**
≥ 2 monovalent doses	18.8 (4.9, 30.7)	**—**	Ref	**—**
2 monovalent doses	**—**	12.8 (-11.4, 31.7)	**—**	Ref
3 monovalent doses	**—**	23.2 (7.1, 36.4)	**—**	11.9 (-10.0, 29.4)
4 monovalent doses	**—**	18.3 (4.1, 30.4)	**—**	6.4 (-14.1, 23.2)
≥ 2 monovalent doses plus a bivalent dose	33.6 (20.8, 44.3)	33.5 (20.7, 44.3)	18.2 (9.4, 26.0)	23.8 (6.0, 38.2)

Analyzed test results were from 17,080 participants (age: 80.9 ± 7.7 years [mean ± standard deviation], 42.3% male). Logistic regression analyses with cluster robust standard errors at the individual level were conducted to estimate the ORs and 95% CIs for testing positive according to the vaccination status. VE was defined as (1 − OR) × 100%

*Abbreviations*: *VE* vaccine effectiveness, *CI* confidence interval, *OR* odds ratio

^a^Adjusted for sex, age group, number of comorbidities, infection history, residential municipality, and test week

^b^This analysis was conducted to assess the effectiveness of receiving ≥ 2 doses of monovalent vaccines compared with unvaccinated status since receiving ≥ 2 doses of monovalent vaccines were required to receive a dose of bivalent vaccines

^c^This analysis was conducted to assess the differences in the effectiveness of completing only primary series (2 monovalent doses), receiving one booster dose (3 monovalent doses), and receiving two booster doses (4 monovalent doses) compared with unvaccinated status since a variety of vaccinated status were assumed during the study period

Table 3 shows the absolute and relative VE of bivalent vaccines against COVID-19 infection according to the interval after receiving the bivalent dose. The absolute and relative VE after a bivalent vaccine dose were highest at 14–20 days after receiving the dose (absolute VE: 40.3% [95% CI: 24.4, 52.8%]; relative VE: 26.5% [95% CI: 11.5, 39.0%]). Then, both absolute and relative VE declined gradually over time, and at ≥ 35 days after the dose, the absolute VE was 36.4% (95% CI: 19.8, 49.5%) and the relative VE was 21.7% (95% CI: 6.1, 34.7%).

**Table 3:** Absolute or relative VE of bivalent vaccines according to interval since bivalent dose

	**n (col. %)**	**Absolute VE**^a^, % (95% CI)	**Relative VE**^a^, % (95% CI)
**Vaccination status**			
Unvaccinated	1,107 (5.5)	Ref	—
≥ 2 monovalent doses	15,189 (76.0)	18.7 (4.8, 30.7)	Ref
≤ 13 days since bivalent dose	905 (4.5)	19.2 (-0.6, 35.1)	0.6 (-17.2, 15.7)
14–20 days since bivalent dose	804 (4.0)	40.3 (24.4, 52.8)	26.5 (11.5, 39.0)
21–34 days since bivalent dose	1,120 (5.6)	37.8 (22.7, 49.9)	23.4 (10.0, 34.9)
≥ 35 days since bivalent dose	873 (4.4)	36.4 (19.8, 49.5)	21.7 (6.1, 34.7)

Analyzed test results were from 17,080 participants (age: 80.9 ± 7.7 years [mean ± standard deviation], 42.3% male). Logistic regression analyses with cluster robust standard errors at the individual level were conducted to estimate the ORs and 95% CIs for testing positive according to the vaccination status. VE was defined as (1 − OR) × 100%

*Abbreviations*: *VE* vaccine effectiveness, *CI* confidence interval, *OR* odds ratio

^a^Adjusted for sex, age group, number of comorbidities, infection history, residential municipality, and test week

Table 4 presents the absolute or relative VE of the bivalent vaccines against infection, stratified by the presence or absence of an infection history. In group without a previous infection, receiving ≥ 2 monovalent doses and a bivalent dose was 33.1% (95% CI: 20.0, 44.0%) more effective than in those without any vaccination and 18.6% (95% CI: 9.9, 26.5%) than receiving ≥ 2 monovalent doses but not receiving a bivalent dose. On the other hand, in having an infection history group, the absolute VE after the bivalent dose was 47.6% (95% CI: -57.1, 82.5%). In addition, receiving a bivalent dose in addition to ≥ 2 monovalent vaccine doses was -0.3% (95% CI: -127.4, 55.8%) more effective than not receiving a bivalent vaccine dose.

**Table 4:** Absolute or relative VE of bivalent vaccines according to infection history

	**Infection history**			
	**No** (*n* = 18,528)		**Yes** (*n* = 1,278)	
	**Absolute VE**^a^, % (95% CI)	**Relative VE**^a^, % (95% CI)	**Absolute VE**^a^, % (95% CI)	**Relative VE**^a^, % (95% CI)
**Vaccination status**				
Unvaccinated	Ref	**—**	Ref	**—**
≥ 2 monovalent doses	17.8 (3.4, 30.0)	Ref	47.7 (-32.9, 79.5)	Ref
≥ 2 monovalent doses plus a bivalent dose	33.1 (20.0, 44.0)	18.6 (9.9, 26.5)	47.6 (-57.1, 82.5)	-0.3 (-127.4, 55.8)

Logistic regression analyses with cluster robust standard errors at the individual level were conducted to estimate the ORs and 95% CIs for testing positive according to the vaccination status. VE was defined as (1 − OR) × 100%

*Abbreviations*: *VE* vaccine effectiveness, *CI* confidence interval, *OR* odds ratio

^a^Adjusted for sex, age group, number of comorbidities, residential municipality, and test week

In auxiliary analyses, at first, among the 3,702 test results obtained in individuals after receiving ≥ 2 monovalent doses and one bivalent vaccine dose, 727 were conducted after receiving BA.1 bivalent vaccines and 2,975 were conducted after receiving BA.4/5 bivalent vaccines. In addition, among the 727 tests conducted after receiving BA.1 bivalent vaccines, 369 were conducted during the period when both BA.1 and BA.4/5 bivalent vaccines were available (between 22 October and 30 December 2022). Compared with the effectiveness of the BA.1 bivalent vaccines against infection, that of the BA.4/5 bivalent vaccines was 2.2% (95% CI: -27.5, 25.0%). Second, the subgroup analysis by test type showed largely similar effectiveness in both types. For instance, compared with receiving ≥ 2 monovalent doses but not a bivalent dose, receiving ≥ 2 monovalent doses plus a bivalent dose was 21.2% (95% CI: 7.0, 33.3%) effective in PCR tests group and 21.9% (95% CI: 11.0, 31.5%) effective in antigen tests group (Supplemental Table 2). Third, in the analysis that focused on the number of received monovalent doses before receiving the bivalent dose, broadly similar effectiveness was observed in received three or four monovalent doses before receiving a bivalent dose category. Received three monovalent doses before receiving a bivalent dose was 34.6% (95% CI: 16.9, 48.4%) effective, and received four monovalent doses was 15.0% (95% CI: 5.3, 23.7%) effective than receiving ≥ 2 monovalent doses but not a bivalent dose (Supplemental Table 3). Fourth, in the analysis that focused on the bivalent vaccines’ brand name, BNT162b2 BA.1 or BA.4/5 vaccines showed similar effectiveness. Receiving BNT162b2 BA.1 vaccine was 17.3% (95% CI: -2.9, 33.5%) and BNT162b2 BA.4/5 vaccine was 17.3% (95% CI: 7.7, 25.8%) effective than receiving ≥ 2 monovalent doses but not a bivalent dose (Supplemental Table 4). Fifth, among the participants who tested positive, 59 and 101 individuals were admitted to a hospital within 7 or 14 days after the date of testing positive, respectively. The prevalence of hospitalization within 14 days of receiving ≥ 2 monovalent doses plus a bivalent dose (2.9%) was lower than that of the unvaccinated group (7.0%) and that of the group that received ≥ 2 monovalent doses but not a bivalent vaccine (3.3%). In addition, similar results were observed for hospitalization within 7 days: the prevalence of hospitalization within 7 days of receiving ≥ 2 monovalent vaccine doses plus a bivalent vaccine dose (1.5%) was lower than that observed in the other groups (Supplemental Table 5).

## Discussion

In this community-based test-negative study, bivalent COVID-19 vaccines were shown to be moderately effective against COVID-19 infection among older adults aged ≥ 65 years in late 2022 in Japan. As shown in Table 2, a bivalent booster dose provided about 20% additional protection for those vaccinated with ≥ 2 monovalent doses. In addition, the bivalent vaccine-conferred protection peaked at 14–20 days after administration and then gradually declined over time. Furthermore, although bivalent vaccines were effective among those without a history of infection, we did not find sufficient evidence of the effectiveness of bivalent vaccines among previously infected older adults.

The findings of this study were generally comparable with those of existing reports [7, 21, 22]. A study from the United States (US) [21] reported that a bivalent booster dose provided 22% (95% CI:15, 29%) additional protection against symptomatic infection among older adults aged ≥ 65 years who had received ≥ 2 monovalent doses. In addition, a study from the Netherlands [7] showed that receiving a bivalent dose in addition to ≥ 2 monovalent doses was 14% (95% CI: 3, 24%) more effective against self-reported infection among older adults aged 60–85 years than was not receiving a bivalent vaccine dose. These studies [7, 21] also assessed the effectiveness of bivalent vaccines by the number of monovalent doses received before the bivalent dose. Both studies found a higher (the US study: 4%; the Dutch study: 10%) protection by the bivalent dose for those who received two monovalent booster doses than one monovalent booster dose. These findings seem to be broadly similar to our findings shown in Table 2 that the difference in relative VE from that of ≥ 2 monovalent doses plus a bivalent dose was higher in that of four monovalent doses (two booster doses) than three monovalent doses (one booster dose) (17.4% vs. 11.9%). Furthermore, in another study from the US [22], the effectiveness of a bivalent booster dose against infection peaked at around 2–4 weeks after vaccination and gradually waned over time thereafter. Although there were some differences in the study methodology, these results were broadly consistent with our finding that the effectiveness of a bivalent booster dose was 18.2% (95% CI: 9.4, 26.0%) among those who had received ≥ 2 monovalent doses, and it gradually waned over time from its peak around 2 weeks after receiving the dose. In addition to these findings in Europe and the US, we believe that our first findings from Eastern countries will advance our understanding of the real-world effectiveness of bivalent COVID-19 vaccines.

In this study, we observed additional protection conferred by bivalent vaccines among participants without a history of infection; however, we did not find enough evidence on the effectiveness of bivalent vaccines among previously infected participants. Similar results were reported in a study from the Netherlands [7]. In the study, whereas a bivalent booster dose provided 14% (95% CI: 1, 25%) additional protection among older adults aged 60–85 years who had not been infected previously, significant effectiveness was not observed among those infected during the Omicron period. Recently, the spring 2023 booster vaccination campaigns were initiated in Japan [23], and the second bivalent booster dose was offered to older adults aged ≥ 65 years who had received their first bivalent dose, regardless of previous infection history. Although older adults are a high-risk population for COVID-19 and vaccination remains the optimal method for safely protecting them from COVID-19, it may be worth considering the appropriate priority group in the target population in future vaccination strategies.

This study had several limitations. First, we used a test-negative design to control for confounding due to differences in health-seeking behavior according to vaccination status. Additionally, we adjusted for demographic and comorbid statuses to address potential confounding factors; however, the possibility of residual confounding by unmeasured factors remains. Second, individuals suspected of having COVID-19 could be tested at medical institutions with public expense support for testing fees during the study period in Japan. Thus, our study participants may have included asymptomatic patients, and our VE estimates should be interpreted as VE against any infection. Third, we assessed the short-term duration of VE of the bivalent vaccines; however, it was not possible to investigate the long-term duration because of the limited time that had passed since initiating bivalent vaccine administration in the study period. Fourth, we merged the results of PCR and antigen tests for analysis, although there was a possibility of misclassification bias due to the differences in diagnostic accuracy between PCR and antigen tests. However, our subgroup analysis by test type observed largely similar results to the main results in both PCR and antigen test groups. Thus, we believe that our results were not greatly affected by merging PCR and antigen tests. As a possible rationale, it was reported that the diagnostic accuracy of antigen tests was moderately agreed with that of PCR tests [24] especially when they were conducted on people with a high possibility of being infected, such as having symptoms [25]. Since our analytical sample was based on the test results conducted in medical institutions, the tests might have been mainly conducted on people who seemed to have a high possibility of being infected; thus, the misclassification bias might be minimal. Fifth, we assessed the effectiveness of bivalent vaccines without distinguishing the vaccine brands in the main analyses although the VE might be differed by the vaccine brands because they have different compositions. We assessed the vaccine brand-specific effectiveness of BNT162b2 BA.1 or BA.4/5 vaccines but could not assess that of mRNA-1273 BA.1 or BA.4/5 vaccines because of uncertainty due to the limited sample size (approximately 2% of our participants who received a bivalent vaccine dose received mRNA-1273 BA.1 or BA.4/5 vaccines). Hence, our estimates of bivalent vaccines’ effectiveness need to be interpreted with caution as they may mostly reflect the effectiveness of BNT162b2 BA.1 or BA.4/5 vaccines. Sixth, our outcome definition was based on the HER-SYS, a reporting system that was used to register infection cases by medical institutions. Although there may be several underreported cases to the HER-SYS, such as when the people did not visit medical institutions for testing, medical doctors were mandated to report the infection cases to the HER-SYS if the tests conducted in the medical institution showed positive results. Hence, we considered that it would be possible to accurately capture the cases at least among the test data conducted in medical institutions using the HER-SYS data. In this context, we believe that we were able to assess the VE by our test-negative study that compared the vaccination status of the participants who received tests in medical institutions. Seventh, our definition of infection history was also based on the HER-SYS data. Given that there may be underreported cases in the HER-SYS, such as because the infection episodes were asymptomatic, several fraction of the participants who actually had infection history might have been classified as not having an infection history category. However, according to our results, the VE was null among the previously infected participants who had at least one infection record in the HER-SYS data. Although this misclassification may lead to underestimation of the VE among the participants without infection history, we believe that our conclusions may not be greatly affected by using the HER-SYS data. Eighth, most participants with a history of infection were infected during the Omicron period (after 2022) (among the 1,470 tests conducted after infection, 1,408 were conducted during the Omicron period). Thus, caution is required when applying our VE estimates of previously infected individuals to those infected during the pre-Omicron period. Ninth, we descriptively assessed VE against hospitalization as a measure of severe COVID-19 among the tested positive participants; however, we did not conduct multivariable analyses because of uncertainty due to the limited number of participants admitted to a hospital after testing positive.

## Conclusions

In conclusion, this study found that bivalent COVID-19 vaccines are expected to have some efficacy against COVID-19 infection in older adults, particularly in those without a history of infection. However, evidence of the effectiveness of bivalent vaccines in previously infected older adults was limited. Our findings provided important insights for the design of future vaccination strategies.

### Supplementary Information

**Additional file 1: Supplemental Table 1.** Number and percentage of each bivalent vaccine product received by the study participants who received ≥2 monovalent doses plus a bivalent dose. **Supplemental Table 2.** Absolute or relative VE of bivalent vaccines against infection by test type. **Supplemental Table 3.** Absolute or relative VE of bivalent vaccines against infection by number of monovalent doses received before receiving a bivalent dose. **Supplemental Table 4.** Absolute or relative VE of bivalent vaccines against infection by vaccine products of bivalent vaccine. **Supplemental Table 5.** Hospitalization within 7 or 14 days from testing positive date according to vaccination status among tested-positive participants. **Supplemental Figure 1.** Trends in number of newly confirmed COVID-19 cases among older adults aged ≥ 65 years in the three municipalities from January 1 to December 31, 2022.

## Data Availability

All data used in this study are not publicly available due to ethical or legal restrictions. For inquiries about the datasets used in this study, please contact the principal investigator of the VENUS Study, Dr. Haruhisa Fukuda, upon reasonable request. Codes used to generate the results in the manuscript may be available from the first author (tamada.yudai97@gmail.com) upon reasonable request. No additional data is available.

## Abbreviations

VE: Vaccine effectiveness
VENUS: Vaccine Effectiveness, Networking, and Universal Safety
HER-SYS: Health Center Real-time Information-sharing System on COVID-19
VRS: Vaccination Record System
LIFE: Longevity Improvement & Fair Evidence
PCR: Polymerase chain reaction
OR: Odds ratio
CI: Confidence interval
STROBE: Strengthening the Reporting of Observational Studies in Epidemiology
SD: Standard deviation

## References

[1] Bobrovitz N, Ware H, Ma X, Li Z, Hosseini R, Cao C (2023). Protective effectiveness of previous SARS-CoV-2 infection and hybrid immunity against the omicron variant and severe disease: a systematic review and meta-regression. Lancet Infect Dis.

[2] Shao W, Chen X, Zheng C, Liu H, Wang G, Zhang B (2022). Effectiveness of COVID-19 vaccines against SARS-CoV-2 variants of concern in real-world: a literature review and meta-analysis. Emerg Microbes Infect.

[3] Canetti M, Barda N, Gilboa M, Indenbaum V, Asraf K, Gonen T (2022). Six-month follow-up after a fourth BNT162b2 vaccine dose. N Engl J Med.

[4] Sane Schepisi M. Early real world evidence on the relative SARS-CoV-2 vaccine effectiveness of bivalent COVID-19 booster doses: a narrative review. 2023. [Preprint]. 10.32388/331ich.

[5] Shrestha NK, Burke PC, Nowacki AS, Simon JF, Hagen A, Gordon SM (2023). Effectiveness of the Coronavirus disease 2019 bivalent vaccine. Open Forum Infect Dis.

[6] Auvigne V, Tamandjou Tchuem CR, Schaeffer J, Vaux S, Parent Du Chatelet I (2023). Protection against symptomatic SARS-CoV-2 infection conferred by the Pfizer-BioNTech Original/BA.4–5 bivalent vaccine compared to the mRNA original monovalent vaccines - a matched cohort study in France. Vaccine.

[7] Huiberts AJ, de Gier B, Hoeve CE, de Melker HE, Hahné SJ, den Hartog G (2023). Effectiveness of bivalent mRNA booster vaccination against SARS-CoV-2 Omicron infection, the Netherlands, September to December 2022. Euro Surveill.

[8] Prime Minister of Japan and His Cabinet. COVID-19 Vaccines. Available: https://japan.kantei.go.jp/ongoingtopics/vaccine.html. Accessed 9 June 2023.

[9] Fukuda H, Maeda M, Murata F (2023). Development of a COVID-19 vaccine effectiveness and safety assessment system in Japan: the VENUS study. Vaccine.

[10] Ministry of Health Labour and Welfare of Japan. Health Center Real-time Information-sharing System on COVID-19 (HER-SYS). Available: https://www.mhlw.go.jp/stf/seisakunitsuite/bunya/0000121431_00181.html. Accessed 9 June 2023.

[11] Digital Agency of Japan. Vaccination record system. Available: https://info.vrs.digital.go.jp/. Accessed 9 June 2023.

[12] Fukuda H, Ishiguro C, Ono R, Kiyohara K. The longevity improvement & Fair evidence (LIFE) study: overview of the study design and baseline participant profile. J Epidemiol. 2022. 10.2188/jea.JE20210513. 10.2188/jea.JE20210513 PMC10319523 35753792

[13] Dean NE, Hogan JW, Schnitzer ME (2021). Covid-19 vaccine effectiveness and the test-negative design. N Engl J Med.

[14] Falsey AR, Frenck RW, Walsh EE, Kitchin N, Absalon J, Gurtman A (2021). SARS-CoV-2 neutralization with BNT162b2 vaccine dose 3. N Engl J Med.

[15] Lopez Bernal J, Andrews N, Gower C, Gallagher E, Simmons R, Thelwall S (2021). Effectiveness of Covid-19 vaccines against the B.1.617.2 (Delta) variant. N Engl J Med.

[16] Lopez Bernal J, Andrews N, Gower C, Robertson C, Stowe J, Tessier E (2021). Effectiveness of the Pfizer-BioNTech and Oxford-AstraZeneca vaccines on covid-19 related symptoms, hospital admissions, and mortality in older adults in England: test negative case-control study. BMJ.

[17] Andrews N, Stowe J, Kirsebom F, Toffa S, Sachdeva R, Gower C (2022). Effectiveness of COVID-19 booster vaccines against COVID-19-related symptoms, hospitalization and death in England. Nat Med.

[18] Altarawneh HN, Chemaitelly H, Ayoub HH, Tang P, Hasan MR, Yassine HM (2022). Effects of previous infection and vaccination on symptomatic omicron infections. N Engl J Med.

[19] Charlson ME, Pompei P, Ales KL, MacKenzie CR (1987). A new method of classifying prognostic comorbidity in longitudinal studies: development and validation. J Chronic Dis.

[20] Andrews N, Tessier E, Stowe J, Gower C, Kirsebom F, Simmons R (2022). Duration of protection against mild and severe disease by Covid-19 vaccines. N Engl J Med.

[21] Link-Gelles R, Ciesla AA, Fleming-Dutra KE, Smith ZR, Britton A, Wiegand RE (2022). Effectiveness of Bivalent mRNA vaccines in preventing symptomatic SARS-CoV-2 infection - increasing community access to testing program, United States, September-November 2022. MMWR Morb Mortal Wkly Rep.

[22] Lin D-Y, Xu Y, Gu Y, Zeng D, Sunny SK, Moore Z (2023). Durability of bivalent boosters against omicron subvariants. N Engl J Med.

[23] Ministry of Health Labour and Welfare of Japan. COVID-19 Vaccine Navi. Available: https://v-sys.mhlw.go.jp/en/flow/. Accessed 9 June 2023.

[24] Lee J, Song J-U, Shim SR (2021). Comparing the diagnostic accuracy of rapid antigen detection tests to real time polymerase chain reaction in the diagnosis of SARS-CoV-2 infection: a systematic review and meta-analysis. J Clin Virol.

[25] Dinnes J, Sharma P, Berhane S, van Wyk SS, Nyaaba N, Domen J (2022). Rapid, point-of-care antigen tests for diagnosis of SARS-CoV-2 infection. Cochrane Database Syst Rev.

