# Supplementary material for: Bivalent mRNA vaccine effectiveness against COVID-19 among older adults in Japan: a test-negative study from the VENUS study
Source: BMC Infect Dis. 2024 Jan 29;24:135. doi: 10.1186/s12879-024-09035-3 (PMC10823731; doi:10.1186/s12879-024-09035-3)
Supplement: Supplementary file 1 — Additional file 1: Supplemental Table 1. Number and percentage of each bivalent vaccine product received by the study participants who received ≥2 monovalent doses plus a bivalent dose. Supplemental Table 2. Absolute or relative VE of bivalent vaccines against infection by test type. Supplemental Table 3. Absolute or relative VE of bivalent vaccines against infection by number of monovalent doses received before receiving a bivalent dose. Supplemental Table 4. Absolute or relative VE of bivalent vaccines against infection by vaccine products of bivalent vaccine. Supplemental Table 5. Hospitalization within 7 or 14 days from testing positive date according to vaccination status among tested-positive participants. Supplemental Figure 1. Trends in number of newly confirmed COVID-19 cases among older adults aged ≥ 65 years in the three municipalities from January 1 to December 31, 2022. [file 12879_2024_9035_MOESM1_ESM.docx]

Supplementary material (online only)

**Title:**

Bivalent mRNA Vaccine Effectiveness against COVID-19 among Older Adults in Japan: A Test-negative Study from the VENUS Study

**Authors:**

Yudai Tamada, Kenji Takeuchi, Taro Kusama, Megumi Maeda, Fumiko Murata, Ken Osaka, and Haruhisa Fukuda

**Table of Contents**

[**Supplemental Table 1.** Number and percentage of each bivalent vaccine product received by the study participants who received ≥2 monovalent doses plus a bivalent dose. 2](#_Toc154753322)

[**Supplemental Table 2.** Absolute or relative VE of bivalent vaccines against infection by test type. 3](#_Toc154753323)

[**Supplemental Table 3.** Absolute or relative VE of bivalent vaccines against infection by number of monovalent doses received before receiving a bivalent dose. 4](#_Toc154753324)

[**Supplemental Table 4.** Absolute or relative VE of bivalent vaccines against infection by vaccine products of bivalent vaccine. 5](#_Toc154753325)

[**Supplemental Table 5.** Hospitalization within 7 or 14 days from testing positive date according to vaccination status among tested-positive participants. 6](#_Toc154753326)

[**Supplemental Figure 1.** Trends in number of newly confirmed COVID-19 cases among older adults aged ≥ 65 years in the three municipalities from January 1 to December 31, 2022. 7](#_Toc154753327)

# **Supplemental Table 1.** Number and percentage of each bivalent vaccine product received by the study participants who received ≥2 monovalent doses plus a bivalent dose.

|  | n (col. %) |
| --- | --- |
| **Vaccine products** |  |
| Pfizer BNT162b2 BA.1 vaccine | 646 (17.5) |
| Pfizer BNT162b2 BA.4/5 vaccine | 2,971 (80.3) |
| Moderna mRNA-1273 BA.1 vaccine | 81 (2.2) |
| Moderna mRNA-1273 BA.4/5 vaccine | 4 (0.1) |

# **Supplemental Table 2.** Absolute or relative VE of bivalent vaccines against infection by test type.

|  | **Test type** | | | | |
| --- | --- | --- | --- | --- | --- |
|  | **PCR** (n = 10,112) | |  | **Antigen** (n = 9,886) | |
|  | **Absolute VE***,  % (95% CI) | **Relative VE***,  % (95% CI) |  | **Absolute VE***,  % (95% CI) | **Relative VE***,  % (95% CI) |
| **Vaccination status** |  |  |  |  |  |
| Unvaccinated | Ref. | **—** |  | Ref. | **—** |
| ≥2 monovalent doses | 5.9  (-21.1, 26.9) | Ref. |  | 25.7  (8.4, 39.7) | Ref. |
| ≥2 monovalent doses plus a bivalent dose | 25.9  (1.3, 44.3) | 21.2  (7.0, 33.3) |  | 42.0  (27.0, 53.8) | 21.9  (11.0, 31.5) |

Abbreviations: VE = vaccine effectiveness; PCR = polymerase chain reaction; CI = confidence interval; OR = odds ratio.

Notes: Logistic regression analyses with cluster robust standard errors at the individual level were conducted to estimate the ORs and 95% CIs for testing positive according to the vaccination status. VE was defined as (1 − OR) × 100%.

* Adjusted for sex, age group, number of comorbidities, infection history, residential municipality, and test week.

# **Supplemental Table 3.** Absolute or relative VE of bivalent vaccines against infection by number of monovalent doses received before receiving a bivalent dose.

|  | **n (col. %)** | **Absolute VE***,  % (95% CI) | **Relative VE***,  % (95% CI) |
| --- | --- | --- | --- |
| **Vaccination status** |  |  |  |
| Unvaccinated | 1,107 (5.5) | Ref. | **—** |
| ≥2 monovalent doses | 15,189 (76.0) | 18.9 (5.0, 30.8) | Ref. |
| 2 monovalent doses  plus a bivalent dose | 33 (0.2) | Not estimated^†^ | Not estimated^†^ |
| 3 monovalent doses  plus a bivalent dose | 692 (3.5) | 46.9 (29.8, 59.8) | 34.6 (16.9, 48.4) |
| 4 monovalent doses  plus a bivalent dose | 2,977 (14.9) | 31.1 (17.5, 42.4) | 15.0 (5.3, 23.7) |

Abbreviations: VE = vaccine effectiveness; CI = confidence interval; OR = odds ratio.

Notes: Logistic regression analyses with cluster robust standard errors at the individual level were conducted to estimate the ORs and 95% CIs for testing positive according to the vaccination status. VE was defined as (1 − OR) × 100%.

* Adjusted for sex, age group, number of comorbidities, infection history, residential municipality, and test week.

^†^ Did not estimated because of uncertainty due to the limited sample size.

# **Supplemental Table 4.** Absolute or relative VE of bivalent vaccines against infection by vaccine products of bivalent vaccine.

|  | **n (col. %)** | **Absolute VE***,  % (95% CI) | **Relative VE***,  % (95% CI) |
| --- | --- | --- | --- |
| **Vaccination status** |  |  |  |
| Unvaccinated | 1,107 (5.5) | Ref. | **—** |
| ≥2 monovalent doses | 15,189 (76.0) | 18.8 (4.9, 30.7) | Ref. |
| ≥2 monovalent doses plus  BNT162b2 BA.1 vaccine | 646 (3.2) | 32.9 (12.7, 48.4) | 17.3 (-2.9, 33.5) |
| ≥2 monovalent doses plus  BNT162b2 BA.4/5 vaccine | 2,971 (14.9) | 32.8 (19.5, 43.9) | 17.3 (7.7, 25.8) |
| ≥2 monovalent doses plus  mRNA-1273 BA.1 vaccine | 81 (0.4) | Not estimated^†^ | Not estimated^†^ |
| ≥2 monovalent doses plus  mRNA-1273 BA.4/5 vaccine | 4 (0.02) | Not estimated^†^ | Not estimated^†^ |

Abbreviations: VE = vaccine effectiveness; CI = confidence interval; OR = odds ratio.

Notes: Logistic regression analyses with cluster robust standard errors at the individual level were conducted to estimate the ORs and 95% CIs for testing positive according to the vaccination status. VE was defined as (1 − OR) × 100%.

* Adjusted for sex, age group, number of comorbidities, infection history, residential municipality, and test week.

^†^ Did not estimated because of uncertainty due to the limited sample size.

# **Supplemental Table 5.** Hospitalization within 7 or 14 days from testing positive date according to vaccination status among tested-positive participants.

|  | **Hospitalization**  **within 14 days*** | |  | **Hospitalization**  **within 7 days^†^** | |
| --- | --- | --- | --- | --- | --- |
|  | **No** | **Yes** |  | **No** | **Yes** |
|  | n (row %) | n (row %) |  | n (row %) | n (row %) |
| **Vaccination status** |  |  |  |  |  |
| Unvaccinated | 174 (93.1) | 13 (7.0) |  | 219 (97.3) | 6 (2.7) |
| ≥2 monovalent doses | 2,276 (96.7) | 77 (3.3) |  | 2,545 (98.3) | 44 (1.7) |
| ≥2 monovalent doses plus a bivalent dose | 373 (97.1) | 11 (2.9) |  | 579 (98.5) | 9 (1.5) |

* A total of 2,924 participants who were tested positive between October 1 and December 16, 2022, were included in the analysis to ascertain the observation period (14 days from the testing positive date).

^†^ A total of 3,402 participants who were tested positive between October 1 and December 23, 2022, were included in the analysis to ascertain the observation period (7 days from the testing positive date).


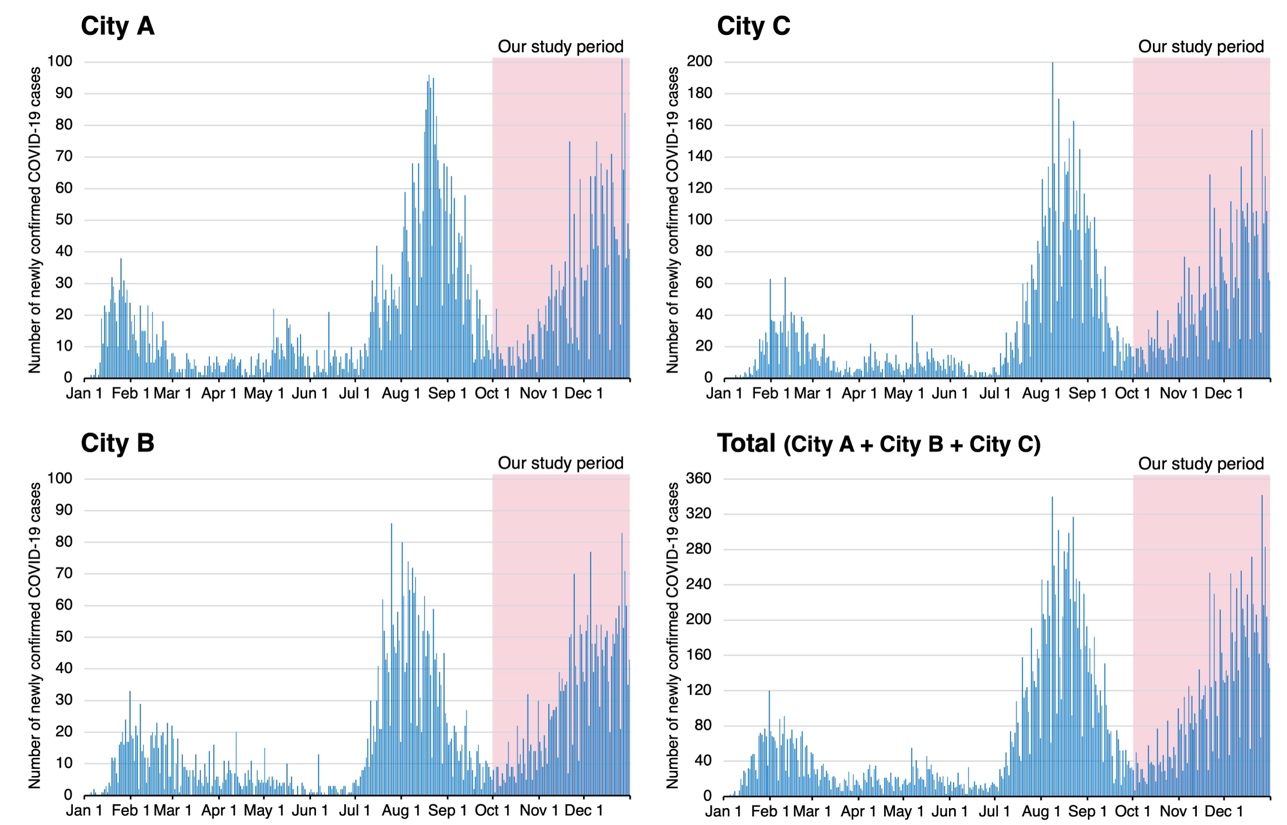


# **Supplemental Figure 1.** Trends in number of newly confirmed COVID-19 cases among older adults aged ≥ 65 years in the three municipalities from 1 January to 31 December 2022.
